# Genome Sequences of Eight *Salmonella enterica* subsp. *enterica* Serovars Isolated from a Single Dairy Farm

**DOI:** 10.1128/genomeA.00082-14

**Published:** 2014-04-24

**Authors:** Bradd J. Haley, Yan Luo, Charles Wang, James Pettengill, Marc Allard, Eric Brown, Jeffrey S. Karns, Jo Ann Van Kessel

**Affiliations:** a Environmental Microbial and Food Safety Laboratory, Beltsville Agricultural Research Center, U.S. Department of Agriculture, ARS, Beltsville, Maryland, USA; b Office of Analytics and Outreach, Division of Public Health Informatics and Analytics, Center for Food Safety and Applied Nutrition, Food and Drug Administration, College Park, Maryland, USA; c Division of Microbiology, Center for Food Safety and Applied Nutrition, Food and Drug Administration, College Park, Maryland, USA

## Abstract

Here, we report draft genome sequences of 26 isolates of *Salmonella enterica* subsp. *enterica*, representing eight serotypes, which were isolated from cows in a Pennsylvania dairy herd, the farm on which they were reared, and the associated off-site heifer-raising facility over an 8-year sampling period.

## GENOME ANNOUNCEMENT

*Salmonella enterica* is a leading cause of gastroenteritis among humans worldwide, with cases estimated at 93.8 million per year and deaths from nontyphoidal *S. enterica* estimated at 155,000 per year (1). Salmonellosis is typically ascribed to the consumption of contaminated poultry, beef, milk, and dairy products, nonanimal products, such as produce, and occasionally, contaminated drinking water (2–4). Poultry and cattle frequently harbor these organisms, sometimes as pathogens resulting in illness or death of the animal, but sometimes as nonpathogenic commensals, thereby presenting a difficult-to-detect public health risk to humans and other animals (5). Further, those people in contact with animals known to be sources of *Salmonella* are at increased risk of infections by these organisms (6).

As part of a long-term longitudinal study of *Salmonella* prevalence in a commercial dairy herd and its associated heifer-rearing facility, samples were collected from feces, composite manure (feces, urine, bedding), trough water, feed, flies caught on the farm, bedding, bulk milk, and milk filters and fecal grab samples, composite manure samples, and water trough samples were also obtained from the farm that raises the postweaned heifers before they are brought back to the farm at which they were born (7, 8). These samples were processed for *S. enterica* using traditional bacteriology methods (7). We selected a subset of *Salmonella* strains from the fecal grab samples for whole-genome sequence analysis.

The 17 *S. enterica* subsp. *enterica* serovar Cerro and two *S. enterica* subsp. *enterica* serovar Kentucky strains were sequenced using 454 Titanium GS FLX+ pyrosequencing (Roche, Branford, CT) to obtain high-quality draft genomes (18 to 23× coverage). The genome contigs were assembled *de novo* with 454 Life Sciences Newbler software package version 2.6 (9). The other *Salmonella* isolates were sequenced using Illumina MiSeq technology (Illumina, Inc., San Diego, CA) to obtain high-quality draft genomes. Genome contigs generated from MiSeq runs were assembled *de novo* with the Velvet software package version 1.2.09 (10). The genome sequencing statistics for these isolates are listed in Table 1. The results describing the evolution and comparative genomics of these isolates will be reported elsewhere.

**TABLE 1:** Genome statistics for the *S. enterica* subsp. *enterica* strains sequenced in this study

*Salmonella enterica* subsp. *enterica* serovar	USDA ID	CFSAN ID	No. of contigs	Genome size (bp)	*N*_50_ contig size (bp)	Accession no.
Cerro	818	CFSAN001330	78	4,736,638	193,703	AOZJ00000000
Cerro	6827	CFSAN001587	71	4,699,129	219,427	AYVG00000000
Cerro	7001	CFSAN001588	81	4,699,438	145,279	AYVF00000000
Cerro	7002	CFSAN001589	65	4,701,086	174,094	AYVE00000000
Cerro	7004	CFSAN001590	74	4,699,430	184,318	AYVD00000000
Cerro	7005	CFSAN001669	97	4,682,990	189,203	AYVC00000000
Cerro	7006	CFSAN001670	75	4,691,976	218,403	AYVB00000000
Cerro	7007	CFSAN001671	76	4,696,980	195,832	AYVA00000000
Cerro	7009	CFSAN001673	79	4,690,427	141,968	AYUZ00000000
Cerro	7010	CFSAN001674	90	4,694,516	140,791	AYUY00000000
Cerro	7020	CFSAN001679	102	4,674,873	140,217	AYUX00000000
Cerro	7021	CFSAN001680	86	4,689,127	162,579	AYUW00000000
Cerro	7022	CFSAN001681	82	4,685,594	151,097	AYUV00000000
Cerro	7032	CFSAN001690	65	4,712,966	211,782	AYUU00000000
Cerro	7033	CFSAN001691	68	4,712,532	222,383	AYUT00000000
Cerro	7034	CFSAN001692	76	4,705,937	131,290	AYUS00000000
Cerro	7036	CFSAN001697	81	4,711,367	142,132	AYUR00000000
Typhimurium var. Copenhagen	084	CFSAN001284	217	5,103,297	50,576	AYVJ00000000
Typhimurium var. 5-	6190	CFSAN004345	227	4,732,039	45,480	AYUO00000000
Enteriditis	3402	CFSAN001333	191	4,912,750	52,316	AYVI00000000
Muenster	5914	CFSAN004344	271	4,836,971	35,024	AYUP00000000
Montevideo	6180	CFSAN004346	258	4,897,282	35,493	AYUN00000000
Give var. 15	117	CFSAN004343	143	4,972,238	76,600	AYUQ00000000
Kentucky	0253	CFSAN001286	108	4,814,097	98,447	AYDR00000000
Kentucky	5349	CFSAN001337	128	4,811,416	84,872	AOYZ00000000
Oranienburg	250	CFSAN001285	138	4,602,146	81,627	AOYM00000000

### Nucleotide sequence accession numbers.

The sequences for the *S. enterica* subsp. *enterica* strains were deposited at NCBI under the accession no. listed in Table 1.
